# Higher frequency of peripheral blood CD103^+^CD8^+^ T cells with lower levels of PD-1 and TIGIT expression related to favorable outcomes in leukemia patients

**DOI:** 10.3389/fimmu.2024.1437726

**Published:** 2024-09-25

**Authors:** Lian Liu, Wenpu Lai, Xiaoling Zhuo, Sihui Chen, Xiaodan Luo, Huo Tan

**Affiliations:** ^1^ Guangzhou Medical University, Guangzhou, China; ^2^ Department of Hematology, Laboratory of Biological Targeting Diagnosis, Therapy and Rehabilitation of Guangdong Higher Education Institutes, The Fifth Affiliated Hospital, Guangzhou Medical University, Guangzhou, China; ^3^ Department of Hematology, First Affiliated Hospital, Jinan University, Guangzhou, China; ^4^ Regenerative Medicine of Ministry of Education, Institute of Hematology, School of Medicine, Jinan University, Guangzhou, China; ^5^ Department of Systems Biomedical Sciences, School of Medicine, Jinan University, Guangzhou, China; ^6^ Flow Morphology Group, Special Testing Technology Center, Guangzhou Huayin Medical Testing Center Special Testing Technology Center, Guangzhou, China

**Keywords:** leukemia, T cell exhaustion, immune checkpoints, tissue resident memory T cells, CD103+CD8+ T cells, PD-1, TIGIT

## Abstract

**Background:**

Leukemia is a prevalent pediatric life-threatening hematologic malignancy with a poor prognosis. Targeting immune checkpoints (ICs) to reverse T cell exhaustion is a potentially effective treatment for leukemia. Tissue resident memory T (T_RM_) cells have been found to predict the efficacy of programmed death receptor-1 inhibitor (anti-PD-1) therapy in solid tumors. However, the IC characteristics of T_RM_ cells in leukemia and their relationship with prognosis remain unclear.

**Methods:**

We employed multi-color flow cytometry to evaluate the frequencies of CD103^+^CD4^+^ and CD103^+^CD8^+^ T cells in the peripheral blood (PB) of patients with acute myeloid leukemia and B-cell acute lymphoblastic leukemia compared to healthy individuals. We examined the expression patterns of PD-1 and T cell immunoreceptor with immunoglobulin and ITIM domain (TIGIT) within the circulating CD103^+^ T cell subsets affected by leukemia. To further elucidate the immunological landscape, we assessed the differentiation status of CD103^+^ T cells across various disease states in patients with leukemia.

**Results:**

Our findings showed a significant increase in the frequency of CD103^+^CD8^+^ T cells in the PB of patients with leukemia who had achieved complete remission (CR) compared to those in the *de novo (DN)* and relapsed/refractory (RR) stages. This increase was accompanied by a notable decrease in the expression levels of PD-1 and TIGIT in CD103^+^CD8^+^ T cells in the CR stage. Additionally, our analysis revealed a higher proportion of CD103^+^CD8^+^ T cells in the central memory (TCM) and effector memory (TEM) subsets of the immune profile. Notably, the proportions of CD103^+^ naïve T cells, CD103^+^ TEM, and CD103^+^ terminally differentiated T cells within the CD8^+^ T cell population were significantly elevated in patients with CR compared to those in the *DN*/RR stages.

**Conclusion:**

The data indicate that circulating higher frequency of CD103^+^CD8^+^ T cells with lower expression of PD-1 and TIGIT are associated with favorable outcomes in patients with leukemia. This suggests a potential role of T_RM_ cells in leukemia prognosis and provides a foundation for developing targeted immunotherapies.

## 1 Introduction

Immune checkpoint inhibitors (ICIs) have revolutionized tumor immunotherapy (1, 2). Programmed death receptor-1 (PD-1), the most well-known inhibitory immune checkpoint (IC), has been approved as a clinical target for cancer therapy (3). T cell immunoreceptor with immunoglobulin and ITIM domain (TIGIT) is an emerging checkpoint receptor that significantly influence the cancer microenvironment (4). Several clinical trials have shown that patients with acute myeloid leukemia (AML) can benefit from programed death receptor-1 inhibitor (anti-PD-1) therapy with minimal adverse reactions. However, some trials have reported less favorable outcomes (5). Several mechanisms contribute to resistance to anti-PD-1/PD-1 ligand 1 (PD-L1) therapy. These include the loss of tumor antigens, interaction with other ICs, activation of oncogenic pathways, epigenetic mutations in key tumor proteins, and changes in metabolism (6). The co-inhibition of TIGIT and PD-1/PD-L1 has shown effectiveness in both theoretical and clinical trials (7).

B-acute lymphoblastic leukemia (B-ALL) and AML are life-threatening pediatric hematologic malignancies. B-ALL is defined as an uncontrolled proliferation of immature B lymphoid cells in the bone marrow, peripheral blood (PB), and other organs (8). High-risk patients may undergo intensive chemotherapy regimens, including corticosteroids, vincristine, and l-asparaginase, with allo-HSCT considered for those in first complete remission (CR) (9). While the event-free survival (EFS) rate for patients with B-ALL has significantly improved to 85% (10), the advent of novel immunotherapies, such as blinatumomab and chimeric antigen receptor T cells have expanded treatment options for patients in the relapsed/refractory (RR) stage. However, it is important to note that these new immunotherapeutic approaches have also been linked to the emergence of new types of relapsed diseases (11, 12). Conversely, acute myeloid leukemia (AML) is relatively uncommon in pediatric populations and is characterized by a diverse range of primary neoplasms originating from myeloid precursor cells, resulting in the accumulation of blasts in the PB, bone marrow, and other tissues (13). The standard therapy for AML involves aggressive induction regimens with anthracyclines and cytarabine and follow-up allo-HSCT for eligible high-risk patients to achieve curative outcomes (13, 14). AML continues to have a poor prognosis, with a 5-year EFS rate of only 50% (15). Furthermore, 10–15% of patients newly diagnosed with AML fail to achieve CR even with intensive chemotherapy, and up to 50% of those who do achieve CR eventually experience relapse (15, 16). Advancements in ALL treatments have outpaced those in AML therapy (17). Patients with RR status typically experience poor outcomes, highlighting the need for novel therapeutic approaches. Recent studies have identified PD-1 and TIGIT expression in exhausted T cells of patients with AML and B-ALL (18, 19). Therefore, investigating PD-1 and TIGIT expression in T cells may offer a new theoretical foundation for the combined use of ICIs.

Tissue resident memory T (T_RM_) cells are a subset of memory T cells that develop and persist predominantly in peripheral nonlymphoid organs, such as the intestines and skin (20–22). They are characterized by the expression of CD103 and CD69. Hobit, Blimp1, Id3, and Runx3 transcription factors are integral to the formation and maintenance of the tissue-resident properties of T_RM_ cells (23, 24). These cells are known for their role in defending organs and tissues against infections (25, 26). Numerous studies have shown that T_RM_ cells exhibit antitumor capabilities in various cancers and are associated with better patient outcomes (27–29). CD8^+^ T_RM_ cells are targeted by ICI (for example, anti-PD-1 and so on) therapy (30, 31). T_RM_ cells within tumor tissues express multiple inhibitory receptors, with elevated PD-1 and TIGIT expression levels on CD8^+^CD103^+^ T cells impairing antitumor function (32, 33). A dual antibody treatment strategy has shown efficacy in ameliorating this depletion activity (34). Our previous study identified a circulating CD103^+^CD8^+^ T cell subset characterized by a high expression of exhaustion-related genes, which was elevated in patients with B-ALL (35). A recent study suggested that CD103^+^CD8^+^ T cells in the bloodstream may be a biomarker for responding to anti-PD-1 regimens in gastric cancer (36). These findings indicate that exhausted circulating T_RM_ cells may be novel therapeutic targets for ICI treatment.

The objective of this study was to investigate the correlation between circulating T_RM_ cells and patient prognosis. This was accomplished by quantifying the proportion of CD103^+^CD8^+^ T cells and assessing the expression levels of PD-1 and TIGIT in the peripheral blood of AML and B-ALL patients with diverse outcomes, utilizing multi-color flow cytometry.

## 2 Materials and methods

### 2.1 PB samples collection

PB samples were collected from patients with AML or B-ALL and healthy individuals (HIs). We obtained 13 samples from patients with leukemia in *DN*/RR states, 9 samples from patients in the CR state, and 20 samples from HIs. Peripheral blood mononuclear cells (PBMCs) were isolated from the blood samples and analyzed using a FACSLyric cytometer (BD Biosciences, San Jose, CA, USA).

### 2.2 Antibody and multi-color flow cytometry

Fluorescence-labeled antibodies used in the study were as follows: CD3-BV786 (clone OKT3, BD), CD4-APC-H7 (clone RPA-T4, BD), CD8-Percp-cy5.5 (clone SK1, BD), CD103 (clone Ber-ACT8, BD), CD45RA-BV510 (clone HI100, BD), CCR7-BV605 (clone G043H7, BioLegend), PD-1-PE-CY7 (clone EH12.1, BD), and TIGIT (clone TgMab-2, BD). Fluorescence-Minus-One Control (FMO) was control background staining and accurate gating, given the low proportion of CD103^+^ T cells in the peripheral blood. PBMCs were first stained with human FC Receptor Binding Inhibitor (Thermo Fisher Scientific) for 15 minutes in PBS, then incubated with antibodies mentioned above at 4°C for 30 mins. Flow cytometry analysis was conducted on washed cells using a FACSLyric cytometer (BD Biosciences), and the data were analyzed using Flowjo 10.6 software.

### 2.3 Statistical analysis

We analyzed differences between groups using the Mann–Whitney *U* test and differences within groups using the nonparametric Wilcoxon matched-pair signed-rank test. Computations were conducted using GraphPad Prism version 8.02 software. A significance level of *p* < 0.05 was considered significant, with levels of significance indicated as *p < 0.05, **p < 0.01, and ***p < 0.001.

## 3 Results

### 3.1 Higher frequency of CD103^+^CD8^+^ T cells in CR leukemia patients

**Figure 1:**
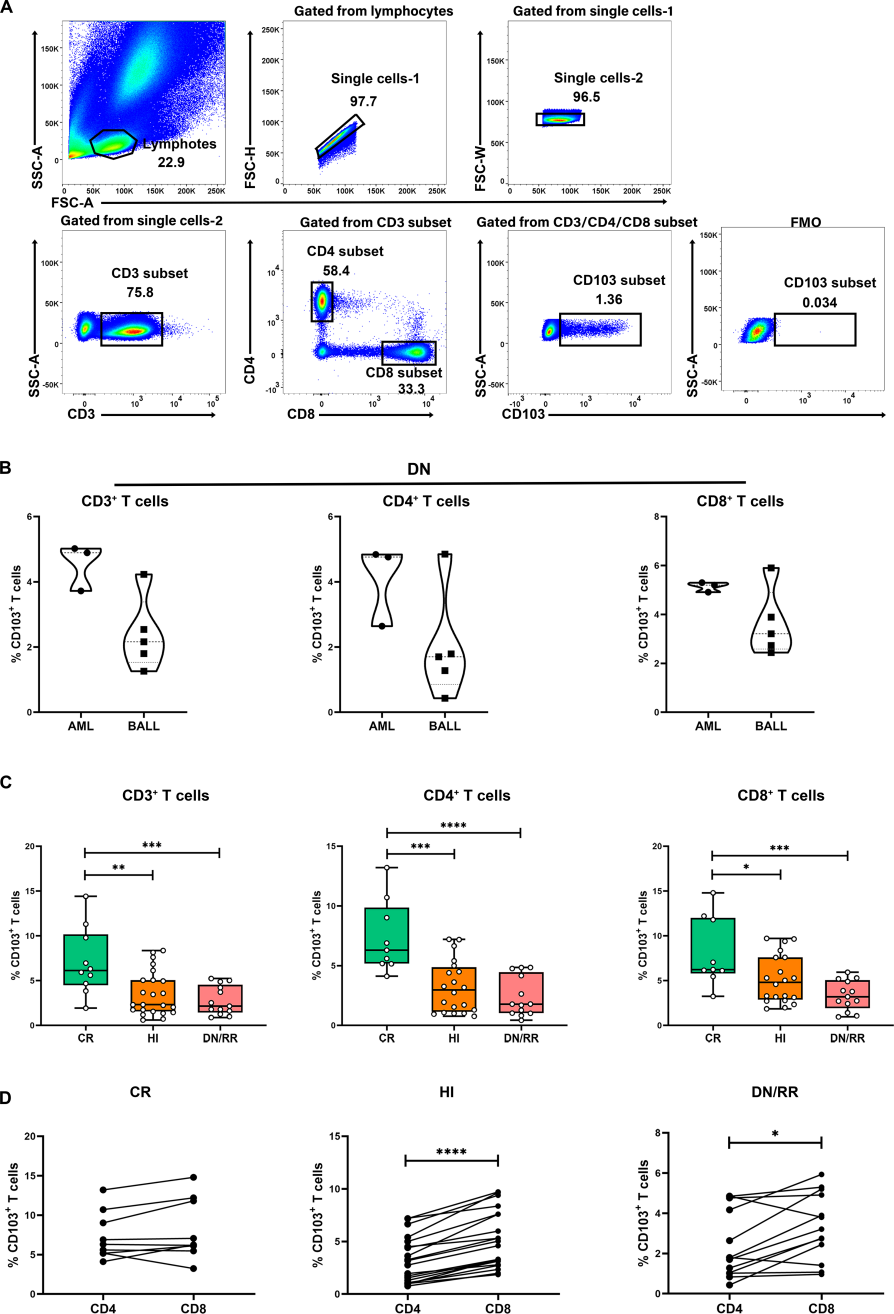
Increased levels of CD103^+^CD3^+^ T cells, CD103^+^CD4^+^ T cells and CD103^+^CD8^+^ T cells in CR patients compared to HI and *DN*/RR patients and the frequencies of CD103^+^CD8^+^ T cells was higher than CD103^+^CD4^+^ T cells of the same PB sample. **(A)**. The gating strategies of CD103^+^CD3^+^ T cells, CD103^+^CD4^+^ T cells and CD103^+^CD8^+^ T cells are shown. **(B)** The ratio of CD103^+^CD3^+^ T cells, CD103^+^CD4^+^ T cells and CD103^+^CD8^+^ T cells in *de novo* AML and *de novo* B-ALL patients. Values are indicated as medians. The significance of differences was calculated using the nonparametric Mann–Whitney *U* test. **(C)**. The frequencies of CD103^+^CD3^+^ T cells, CD103^+^CD4^+^ T cells and CD103^+^CD8^+^ T cells in CR, HIs and *DN*/RR patients. (CR *n* = 9, HI *n* = 20, *DN*/RR *n* = 13). Values are indicated as medians. The significance of differences was calculated using the nonparametric Mann–Whitney *U* test. **(D)** Frequencies of CD103^+^CD4^+^ T cells and CD103^+^CD8^+^ T cells of the same peripheral blood sample. The significance of differences was calculated using the nonparametric Wilcoxon matched-pairs signed-rank test. (*p < 0.05, **p < 0.01, ***p < 0.001, ****p < 0.0001) CR, complete remission; HIs, healthy individuals; *DN*/RR, *de novo* and relapsed/refractory. P values < 0.05 were considered statistically significant.

**Figure 2:**
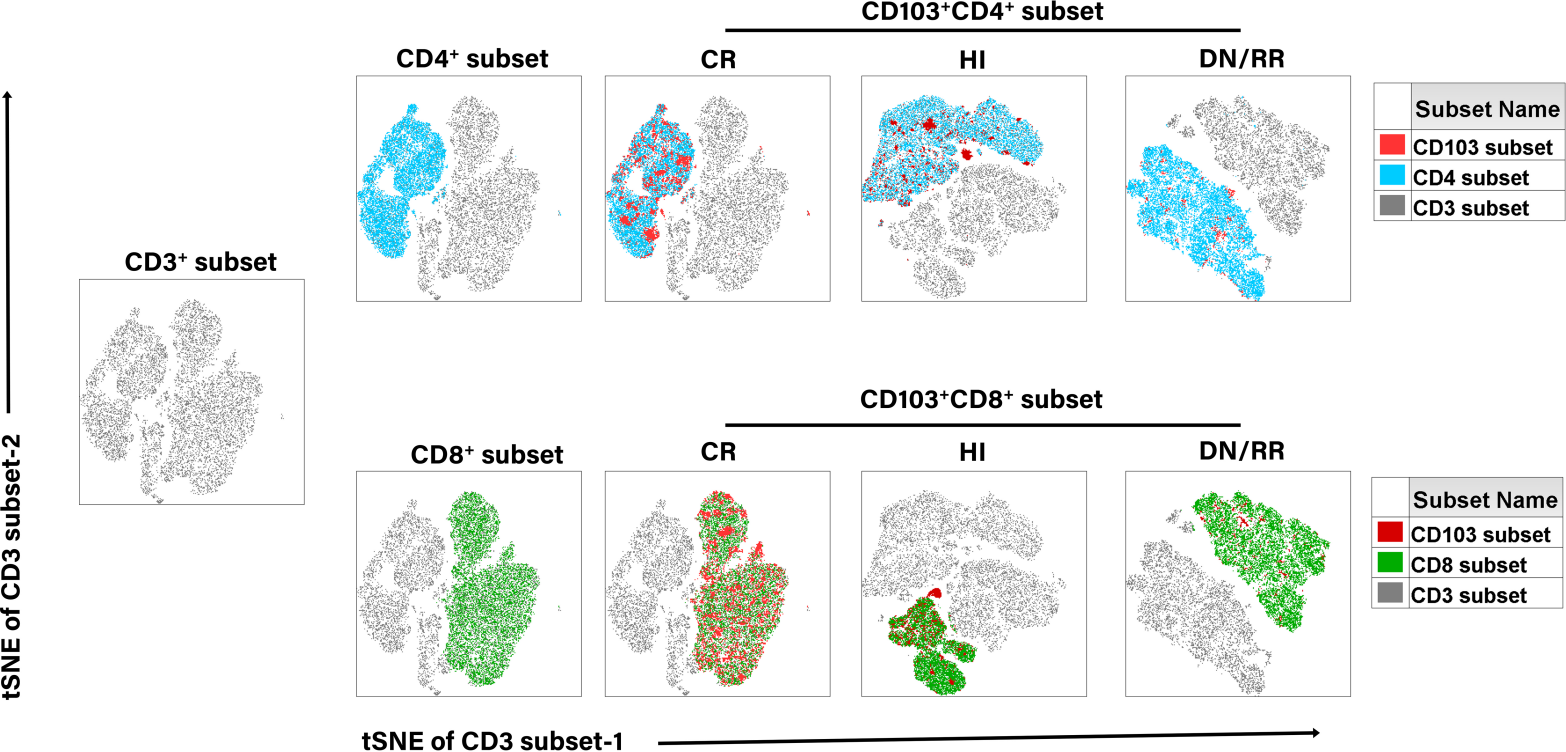
T-distributed random neighbor embedded (tSNE) analysis of the distribution of CD103^+^ T cells in CD3^+^, CD4^+^, and CD8^+^ T cells in CR, HIs and *DN*/RR patients.

We initially evaluated CD103 expression levels among CD3^+^, CD4^+^, and CD8^+^ T cells in PB (**Figure 1A**). There were no significant differences in the proportions of CD103^+^CD3^+^ T cells, CD103^+^CD4^+^ T cells, and CD103^+^CD8^+^ T cells between patients with *DN*-AML and those with *DN*-B-ALL (**Figure 1B**). Therefore, subsequent analyses pooled patients with AML and B-ALL. The frequencies of CD103^+^CD3^+^, CD103^+^CD4^+^, and CD103^+^CD8^+^ T cells were significantly higher in patients with leukemia in CR than in HIs and *DN*/RR. The percentage of CD103^+^CD8^+^ T cells in patients with leukemia in the *DN*/RR states was lower than in HIs. However, this was not statistically significant (*p =* 0.072, **Figure 1C**). Furthermore, in HIs and patients with leukemia in *DN*/RR states, CD103^+^CD8^+^ T cells were more prevalent than CD103^+^CD4^+^ T cells within the same peripheral blood sample. In patients with leukemia in the CR state, no significant difference was observed between CD103^+^CD4^+^ andCD103^+^CD8^+^ T cells from the same samples (**Figure 1D**). The t-stochastic neighbor embedding analysis of CD3^+^ T cells from HIs and patients with leukemia in CR, *DN*/RR states is shown in **Figure 2**.

### 3.2 Elevated PD-1 and TIGIT phenotypes in CD103^+^ T Cells in DN/RR patients

**Figure 3:**
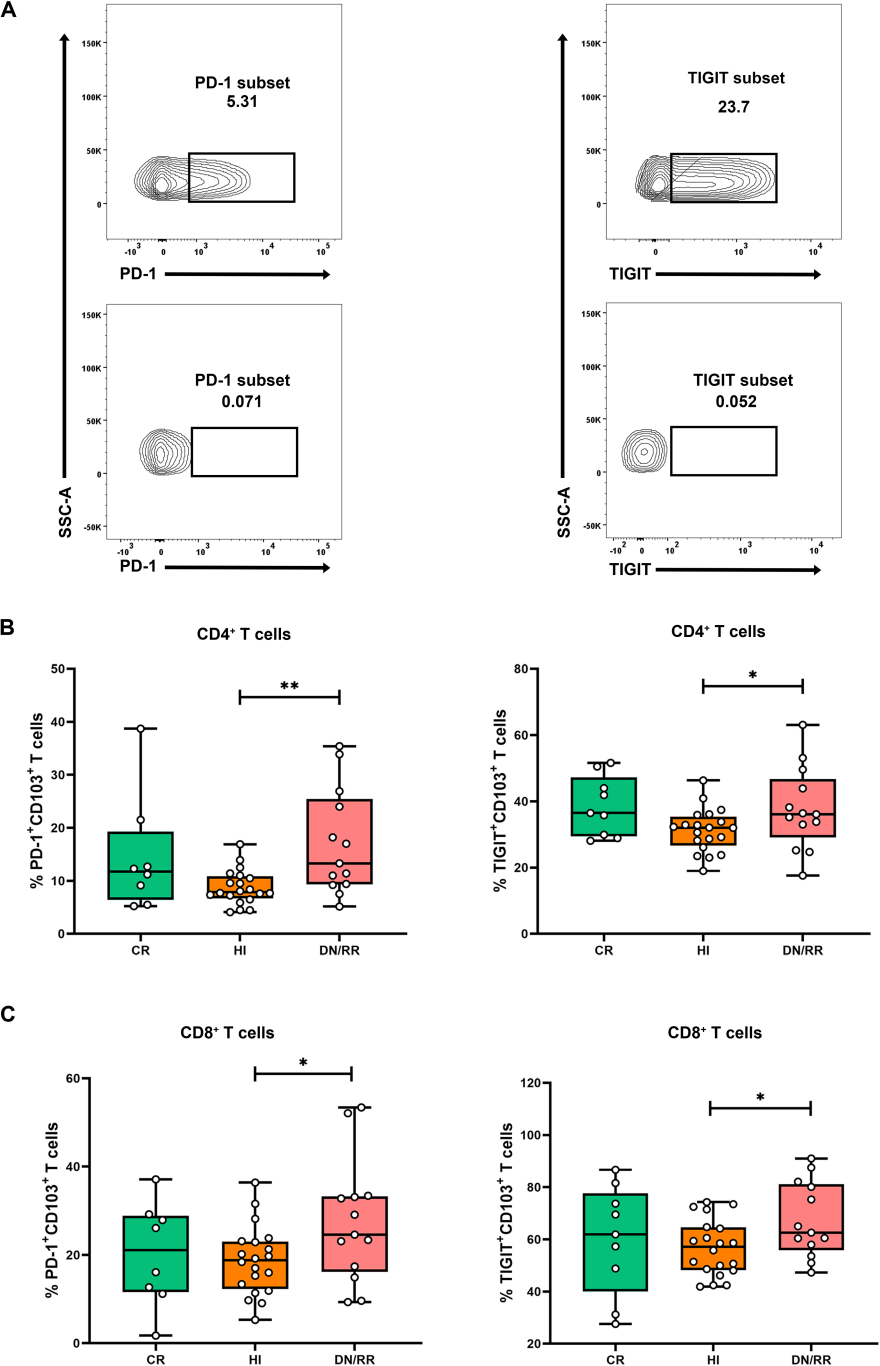
Higher frequencies of PD-1^+^CD103^+^CD4^+^ T cells, PD-1^+^CD103^+^CD8^+^ T cells, TIGIT^+^CD103^+^CD4^+^ T cells and TIGIT^+^CD103^+^CD8^+^ T cells in *DN*/RR patients compared with HI. **(A)** Flow cytometry analysis of the ration of the frequency of PD-1 and TIGIT in CD103^+^ T cells. **(B)** The percentages of PD-1^+^CD103^+^CD4^+^ T cells, PD-1^+^CD103^+^CD8^+^ T cells in CR, HIs and *DN*/RR patients. (CR *n* = 8, due to one of the CR patients using nivolumab treatment regimen, the PD-1 expression frequency cannot be detected.) **(C)** The percentages of TIGIT^+^CD103^+^CD4^+^ T cells, TIGIT^+^CD103^+^CD8^+^ T cells in CR, HIs and *DN*/RR patients. Values are indicated as medians. The significance of differences was calculated using the nonparametric Mann–Whitney *U* test. (*p < 0.05, **p < 0.01).

To characterize the exhaustion characteristics of the CD103^+^ T cell subset, we analyzed the expression levels of PD-1 and TIGIT in CD103^+^CD4^+^ and CD103^+^CD8^+^ T cells (**Figure 3A**). Patients in *DN*/RR states exhibited significantly higher proportions of PD-1^+^CD103^+^CD4^+^ and PD-1^+^CD103^+^CD8^+^ T cells than HIs. There were no statistically significant differences in the proportions of PD-1^+^CD103^+^CD4^+^ T cells and PD-1^+^CD103^+^CD8^+^ T cells between CR patients and HIs or between CR and *DN*/RR patients. Furthermore, patients with DN/RR displayed higher frequencies of TIGIT^+^CD103^+^CD4^+^ T cells and TIGIT^+^CD103^+^CD8^+^ T cells than those with HIs. A trend toward a higher level of TIGIT^+^CD103^+^CD4^+^ T cells was observed in CR patients than in HIs, although this did not reach statistical significance (*p* = 0.0617, **Figures 3B, C**). In summary, elevated PD-1 and TIGIT expression levels in CD103^+^ T cells were observed in patients with leukemia in the *DN* and RR states.

### 3.3 Differentiation characteristics of CD103^+^CD8^+^ T cells in leukemia patients

**Figure 4:**
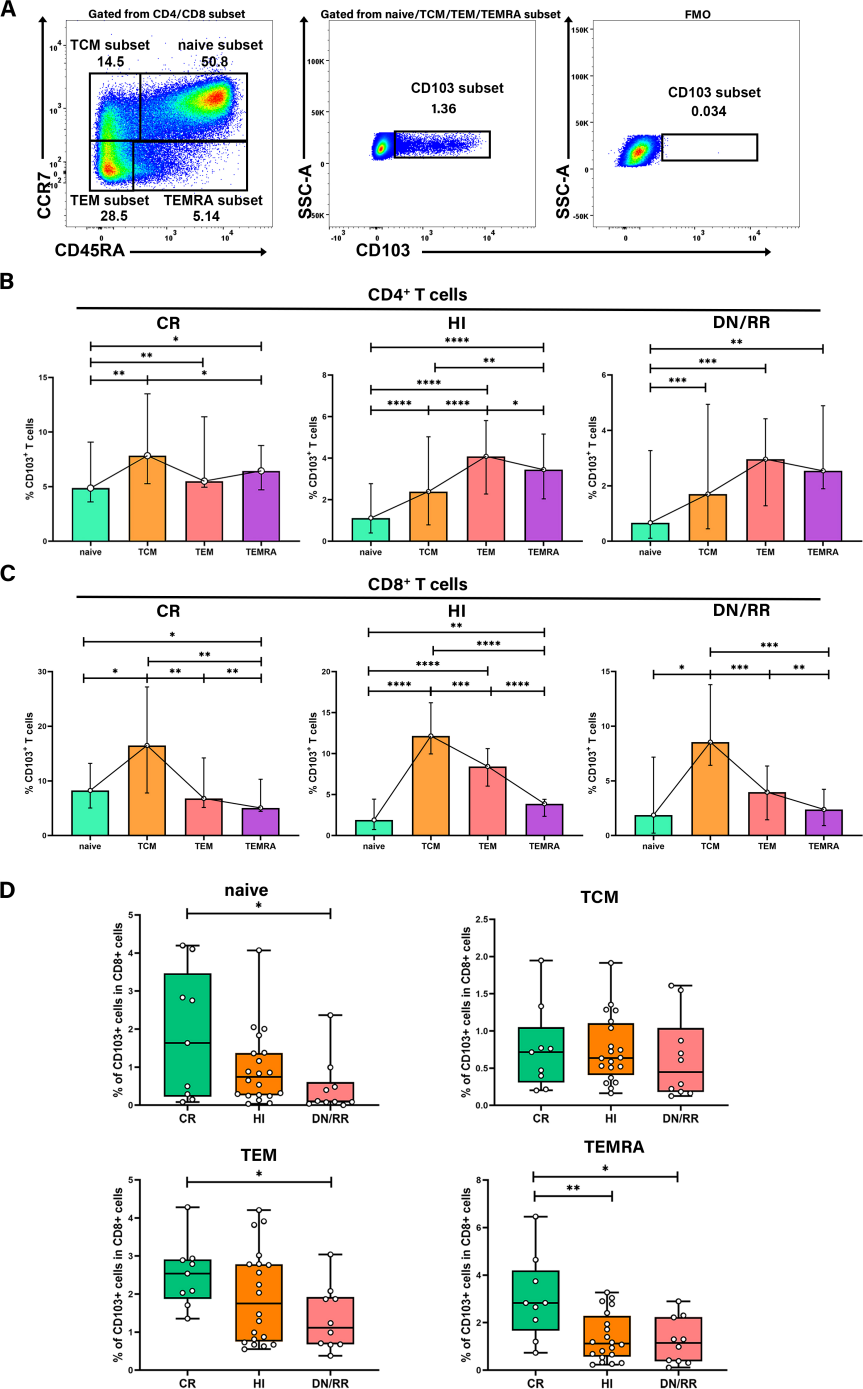
The frequency of CD103^+^CD8^+^ T cells was higher in TCM and TEM subsets and the ratio of CD103^+^ naïve T cells, CD103^+^ TEM T cells, and CD103^+^ TEMRA T cells in CD8^+^ T cells were higher in CR patients than in *DN*/RR patients. **(A)** Flow cytometry analysis of the CD103^+^ T cells in different differentiation subsets. **(B, C)** The percentages of CD103^+^CD4^+^ T cells, CD103^+^ CD8^+^ T cells in each differentiation subset. Values are indicated as medians. The significance of differences was calculated using the nonparametric Wilcoxon matched-pairs signed-rank test. **(D)** The proportion of CD103^+^ naïve T cells, CD103^+^ TEM T cells, and CD103^+^ TEMRA T cells in CD8^+^ T cells of CR, HIs and *DN*/RR patients. Values are indicated as medians. The significance of differences was calculated using the nonparametric Mann–Whitney *U* test. (*p < 0.05, **p < 0.01, ***p < 0.001, ****p < 0.0001).

We analyzed the differentiation status of T cells using the naïve T cell marker CD45RA and the chemokine receptor CCR7. This analysis categorized T cells as naïve (CD45RA^+^CCR7^+^), central memory (TCM, CD45RA^−^CCR7^+^), effector memory (TEM, CD45RA^−^CCR7^−^), and terminally differentiated (TEMRA, CD45RA^+^CCR7^−^) subsets. We then examined the distribution of CD103^+^ T cells across these differentiation subsets (**Figure 4A**). CD103^+^CD4^+^ T cells level was higher in the TEM and TEMRA subsets, with a lower level found in the naïve subset than in the other differentiation subsets. This pattern was not statistically significant among patients with leukemia in the CR state (**Figure 4B**). However, for CD103^+^CD8^+^ T cells, the highest level was observed in the TCM subset, followed by the TEM subset. The naïve and TEMRA subsets exhibited the lowest level of CD103^+^CD8^+^ T cells in the CR, HIs, *DN*/RR groups (**Figure 4C**). To avoid variations in the distribution of naïve, TCM, TEM, and TEMRA subsets among individuals, we analyzed the levels of CD103^+^ naïve, TCM, TEM, and TEMRA T cells within the total CD8^+^ T cells. The levels of CD103^+^ naïve, TEM, and TEMRA T cells were higher in the CR than in the *DN*/RR groups. Furthermore, the CR groups exhibited elevated levels of CD103^+^ TEMRA T cells within CD8^+^ T cells compared to HIs (**Figure 4D**). No significant differences were found in the levels of CD103^+^ TCM T cells within CD8^+^ T cells among the CR, HI, *DN*/RR groups. The elevated level of CD103^+^CD8^+^ T cells in the TCM and TEM subsets indicates that T_RM_ cells in PB may promptly respond to secondary antigen exposure and exert antitumor functions similar to TCM and TEM cells. Moreover, CD103^+^ naïve, TEM, and TEMRA T cells within CD8^+^ T cells may contribute to CR outcomes in patients with AML and B-ALL.

### 3.4 The distribution of CD103^+^ T cells in AML and B-ALL patients respectively

**Figure 5:**
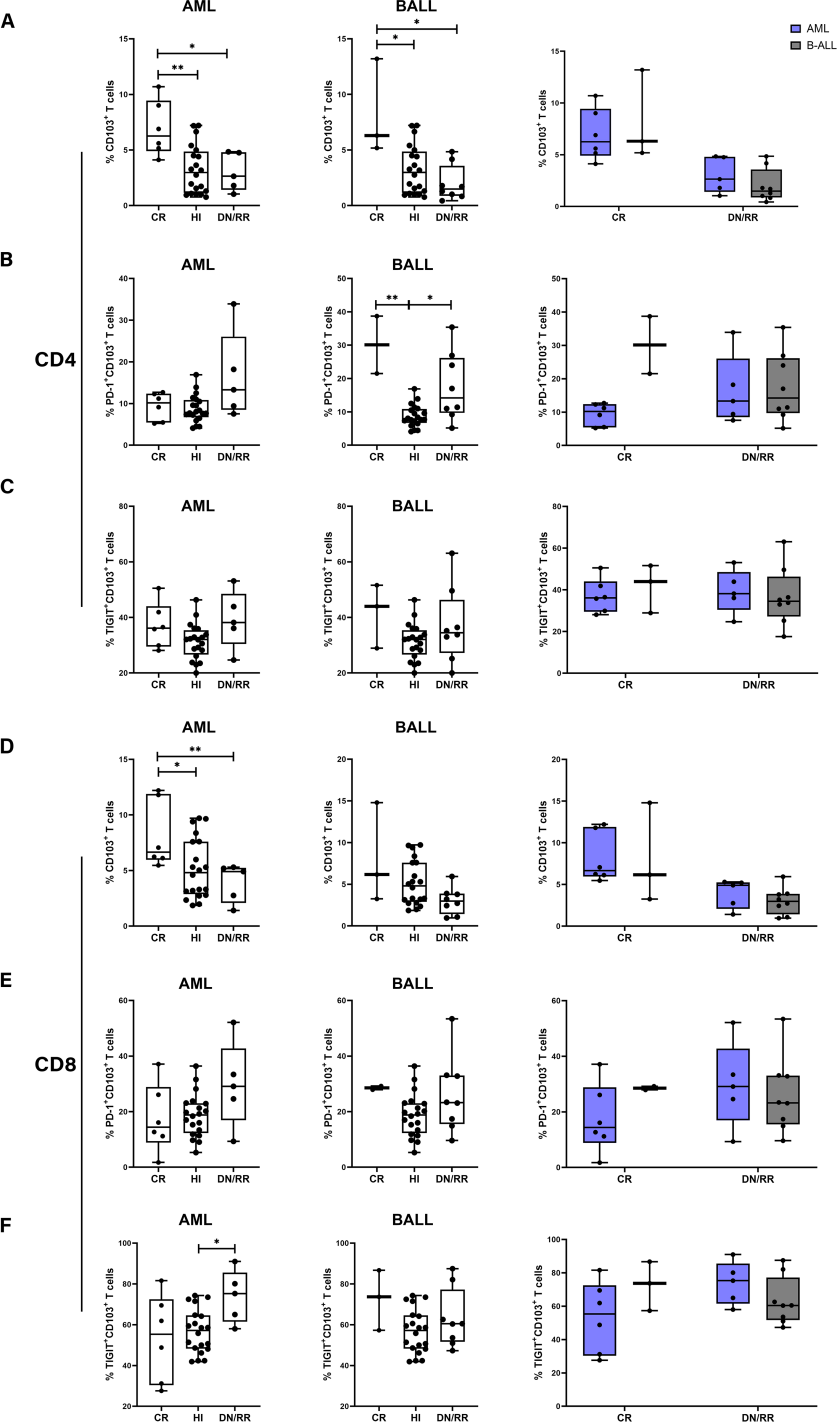
The proportion of CD103^+^CD4^+^ T cells and CD103^+^CD8^+^ T cells in CR patients compared to HI and *DN*/RR patients and the frequencies of exhausted phenotype CD103^+^ cells was higher in *DN*/RR patients with AML and B-ALL respectively and comparison between AML and B-ALL patients. **(A, D)** The frequencies of CD103^+^CD4^+^ T cells and CD103^+^CD8^+^ T cells in CR, HIs and DN/RR patients with AML and B-ALL respectively and comparison between AML and B-ALL patients. (AML CR *n* = 6, *DN*/RR *n* = 5; B-ALL CR *n* = 3, *DN*/RR *n* = 8). **(B, E)** The percentages of PD-1^+^CD103^+^CD4^+^ T cells, PD-1^+^CD103^+^CD8^+^ T cells in CR, HIs and *DN*/RR patients with AML and B-ALL respectively and comparison between AML and B-ALL patients. (B-ALL CR *n* = 2, due to one of the CR patients using nivolumab treatment regimen, the PD-1 expression frequency cannot be detected.) **(C, F)** The percentages of TIGIT^+^CD103^+^CD4^+^ T cells, TIGIT^+^CD103^+^CD8^+^ T cells in CR, HIs and *DN*/RR patients with AML and B-ALL respectively and comparison between AML and B-ALL patients. Values are indicated as medians. The significance of differences was calculated using the nonparametric Mann–Whitney *U* test. (*p < 0.05, **p < 0.01).

We analyzed the distribution and exhaustion of CD103^+^ T cells in the PB of patients with AML and B-ALL respectively. In patients with AML, the frequencies of CD103^+^CD4^+^ and CD103^+^CD8^+^ T cells were higher in the CR group than in the HIs, *DN*/RR groups. No significant differences were observed between the HIs, *DN*/RR groups. In patients with B-ALL, a similar increase in CD103^+^CD4^+^ T cells was observed in the CR group compared to the HIs, *DN*/RR groups. Furthermore, the level of CD103^+^CD8^+^ T cells tended to decrease in the *DN*/RR groups compared to that in the CR and HIs groups, although the difference was not statistically significant (DN/RR *vs.* CR, *p* = 0.0850; *DN*/RR *vs.* HIs, *p* = 0.0750). No significant differences were observed between patients with AML and B-ALL (**Figures 5A, D**). The proportion of PD-1^+^CD103^+^CD4^+^ T cells, indicative of an exhausted phenotype, was higher in CR, *DN*/RR patients with B-ALL than in HIs. Similarly, the expression level of TIGIT^+^CD103^+^CD8^+^ T cells, also defined as exhausted phenotype, increased compared to the HIs. No further significant differences were identified among these groups (**Figures 5B, C, E, F**).

## 4 Discussion

While PD-1/PD-L1 inhibitors have proven effective in treating solid tumors, their activity in AML and B-ALL has been less significant (37). Identifying novel and specific targets may be crucial to addressing this disparity. In our previous study, we identified circulating T_RM_-like cells with a relatively high level of exhaustion genes in patients with B-ALL using single-cell sequencing (35), indicating that T_RM_ cells could be a potential new target for ICIs. However, in this study, we investigated the differences in CD103^+^CD8^+^ T cells between patients with leukemia in the CR state and those with *DN*/RR states.

First, a study reported that a subset of T_RM_ cells, characterized by their recognition of ovarian cancer antigens, effectively inhibited tumor growth compared to CD103^-^CD8^+^ T cells (38). This finding is corroborated by RNA sequencing data indicating that CD103^+^CD8^+^ T cells possess an enhanced cytotoxic profile and exhibit a gene signature associated with interferon-γ (IFN-γ) production, distinguishing them from other CD8^+^ T cells (39). Given their dual ability to recognize tumor cells, inhibit growth, secrete IFN-γ, and perform cytotoxic functions, we suggest that these cells may be tumor-specific and potentially associated with favorable clinical outcomes. Our findings align with previous studies on head and neck cancer, demonstrating that patients who achieved remission following anti-PD-1 monoclonal antibody therapy exhibited a significant increase in CD103^+^CD8^+^ T cells in their pretreatment biopsy tissues compared to those who did not achieve remission (30). The higher expression level of CD103^+^CD8^+^ T cells in PB from patients in CR, as opposed to those in *DN*/RR, supports the antitumor function of circulating T_RM_ cells in patients with acute leukemia.

Second, patients in the *DN*/RR states showed significantly higher levels of exhausted phenotype CD103^+^CD8^+^ T cells than HIs. A recent study systematically outlined the dynamics of T_RM_ cells in response to pembrolizumab therapy. It was observed that patients who achieved remission following treatment predominantly exhibited an increase in cytotoxic T_RM_ cells, characterized by the expression of granzyme H, granzyme K, SLAM family member 7, and eomesodermin, alongside reduced exhaustion markers. Conversely, tissues from patients who did not achieve remission exhibited amplified T_RM_ cells with increased exhaustion profiles, characterized by the expression of PDCD1, T-cell immunoglobulin and mucin-domain containing-2, TIGIT, cytotoxic T-lymphocyte-associated protein 4, and thymocyte selection-associated high mobility group box protein (30). This indicates that besides the decrease in CD103^+^CD8^+^ T cells, diminished antileukemia function also contributes to the occurrence and progression of AML and B-ALL. TIGIT, an emerging IC molecule, is gaining attention in hematological malignancies and is seen as a potential therapeutic target (40, 41). Studies have demonstrated that TIGIT inhibits CD8^+^ T cells function in the immune response against tumors and viruses (42). In our previous research, we found that the TIGIT expression of CD8^+^ T cells in the bone marrow and peripheral blood of AML patients was higher than that in HIs (43), suggesting that TIGIT may play an important role in T cell dysfunction in leukemia patients. Dual blockade of the PD-1 and TIGIT enhance the anti-tumor ability of CD8^+^ T cells (44). Consistent with a recent study on endometrial cancer (34), high PD-1 and TIGIT expression levels indicate that CD103^+^CD8^+^ T cells are promising targets for ICIs. Additionally, another study identified the expression of immunosuppressive receptors on the surface of T_RM_ cells, where PD-1 and TIM-3 double-positive cells show functional impairment (45, 46).

Third, among the naïve, TCM, TEM, and TEMRA subsets, the TCM and TEM subsets exhibited higher expression levels of CD103^+^CD8^+^ T cells. This finding is corroborated by another study on circulating T_RM_ cells (36) suggesting that upon re-exposure to tumor antigens, CD103^+^CD8^+^ T cells differentiate into effector cells, such as TCM and TEM cells, thereby exerting an antitumor function. Reports indicate that CD8^+^ memory T cells are the predominant phenotype expanded during anti-PD-1 blockade therapy (47), potentially explaining why the CD103^+^CD8^+^ T cell subset is a biomarker for ICI efficacy. Due to variations in proportions of naïve/TCM/TEM/TEMRA subgroups among individuals, we conducted a detailed analysis of the frequencies of CD103^+^ naïve, TCM, TEM, and TEMRA T cells within CD8^+^ T cells across patients in CR, *DN*/RR states and HIs. The expression levels of CD103^+^ naïve, TEM, and TEMRA within CD8^+^ T cells were higher in patients in the CR state than in HIs and patients in those in *DN*/RR states, suggesting that increased expression levels of CD103^+^ T cells in these subsets may facilitate CR. Studies have shown that CCR7, known for mediating T cell entry into secondary lymphoid organs (48), is crucial in forming CD103^+^CD8^+^ T cells and its tumor-clearing capabilities (49). We also suggest that the increase in CD103^+^ naïve T cells in patients in the CR state is a significant factor in achieving CR.

Finally, the differences in CD103^+^ T cells in the PB of patients with AML and B-ALL were examined. We also found higher expression levels of CD103^+^CD4^+^ and CD103^+^CD8^+^ T cells in patients with AML or B-ALL in the CR state than in the *DN*/RR states and HIs. Similarly, a higher expression level of the exhausted phenotype was observed in CD103^+^ T cells in patients in *DN*/RR states than in HIs. Nevertheless, this study has some limitations. The low incidence of hematologic diseases in pediatric populations has constrained our ability to identify significant differences in the expression levels of CD103^+^CD3^+^ T cells, CD103^+^CD4^+^ T cells, and CD103^+^CD8^+^ T cells between patients in *DN*/RR states and HIs, primarily due to small sample sizes. This limitation also affected the data analysis for patients with AML and B-ALL. Therefore, further research with larger cohort sizes is needed to thoroughly analyze the distribution patterns and exhaustion phenotypes of circulating T_RM_ cells.

In summary, these findings indicate that a higher frequency of CD103^+^CD8^+^ T cells in PB and lower expression levels of PD-1^+^CD103^+^CD8^+^ T cells and TIGIT^+^CD103^+^CD8^+^ T cells are associated with a higher likelihood of achieving CR outcomes in AML and B-ALL. A higher level of CD103^+^CD8^+^ T cells was observed within the TCM and TEM subsets. Moreover, the levels of CD103^+^ naïve, TEM, and TEMRA T cells within the CD8^+^ T cells of patients in the CR state were higher than those in the *DN* and RR states. Identifying CD103^+^CD8^+^ T cells as a potential predictive factor for favorable prognosis requires further validation, which may necessitate larger sample sizes. In addition, selecting biomarkers suitable for immune checkpoint drugs in AML and B-ALL patients, and even predicting the efficacy of adoptive T cell therapy, including chimeric antigen receptor T (CAR-T) cell therapy. Further study is necessary to validate CD103^+^CD8^+^ T cells as a promising novel target for enhancing current cancer immunotherapies in pediatric patients.

## Data Availability

The original contributions presented in the study are included in the article/supplementary material. Further inquiries can be directed to the corresponding authors.
